# (*E*)-4-Chloro-2-[(2-hydroxy­phenyl)­iminometh­yl]phenol

**DOI:** 10.1107/S160053681001233X

**Published:** 2010-04-14

**Authors:** Naser Eltaher Eltayeb, Siang Guan Teoh, Suchada Chantrapromma, Hoong-Kun Fun

**Affiliations:** a School of Chemical Sciences, Universiti Sains Malaysia, 11800 USM, Penang, Malaysia; b Crystal Materials Research Unit, Department of Chemistry, Faculty of Science, Prince of Songkla University, Hat-Yai, Songkhla 90112, Thailand; c X-ray Crystallography Unit, School of Physics, Universiti Sains Malaysia, 11800 USM, Penang, Malaysia

## Abstract

The title compound, C_13_H_10_ClNO_2_, exists in a *trans* configuration about the central C=N bond. The two benzene rings are almost coplanar, making a dihedral angle of 2.48 (10)°. An intra­molecular O—H⋯N hydrogen bond generates an *S*(6) ring motif. In the crystal structure, O—H⋯O hydrogen bonds link the mol­ecules into chains along [101]. Short C⋯Cl contacts [3.584 (2)–3.646 (2) Å] are observed. A short intramolecular C—H⋯O contact occurs.

## Related literature

For bond-length data, see: Allen *et al.* (1987 ▶). For hydrogen-bond motifs, see: Bernstein *et al.* (1995 ▶). For background to Schiff bases and their applications, see: Dao *et al.* (2000 ▶); Eltayeb & Ahmed (2005*a* ▶,*b* ▶); Karthikeyan *et al.* (2006 ▶); Sriram *et al.* (2006 ▶); Wei & Atwood (1998 ▶). For related structures, see: Eltayeb *et al.* (2007*a* ▶,*b* ▶); Pu (2008 ▶). For the stability of the temperature controller used in the data collection, see: Cosier & Glazer (1986 ▶).
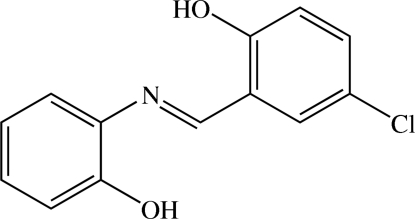


## Experimental

### 

#### Crystal data

C_13_H_10_ClNO_2_
*M* *_r_* = 247.67
Monoclinic, 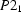

*a* = 4.6681 (9) Å
*b* = 18.509 (3) Å
*c* = 6.2118 (11) Å
β = 90.980 (4)°
*V* = 536.63 (17) Å^3^
*Z* = 2
Mo *K*α radiation
μ = 0.34 mm^−1^
*T* = 100 K
0.55 × 0.14 × 0.07 mm

#### Data collection

Bruker APEX Duo CCD area detector diffractometer
Absorption correction: multi-scan (*SADABS*; Bruker, 2009 ▶) *T*_min_ = 0.834, *T*_max_ = 0.976
4593 measured reflections
2680 independent reflections
2569 reflections with *I* > 2σ(*I*)
*R*_int_ = 0.023

#### Refinement

*R*[*F* ^2^ > 2σ(*F* ^2^)] = 0.034
*wR*(*F* ^2^) = 0.119
*S* = 1.20
2680 reflections
154 parameters
1 restraint
H-atom parameters constrained
Δρ_max_ = 0.47 e Å^−3^
Δρ_min_ = −0.47 e Å^−3^
Absolute structure: Flack (1983 ▶), 1125 Friedel pairs
Flack parameter: −0.03 (7)

### 

Data collection: *APEX2* (Bruker, 2009 ▶); cell refinement: *SAINT* (Bruker, 2009 ▶); data reduction: *SAINT*; program(s) used to solve structure: *SHELXTL* (Sheldrick, 2008 ▶); program(s) used to refine structure: *SHELXTL*; molecular graphics: *SHELXTL*; software used to prepare material for publication: *SHELXTL* and *PLATON* (Spek, 2009 ▶).

## Supplementary Material

Crystal structure: contains datablocks global, I. DOI: 10.1107/S160053681001233X/is2534sup1.cif

Structure factors: contains datablocks I. DOI: 10.1107/S160053681001233X/is2534Isup2.hkl

Additional supplementary materials:  crystallographic information; 3D view; checkCIF report

## Figures and Tables

**Table 1:** Hydrogen-bond geometry (Å, °)

*D*—H⋯*A*	*D*—H	H⋯*A*	*D*⋯*A*	*D*—H⋯*A*
O1—H1*O*1⋯O2^i^	0.82	1.74	2.553 (2)	169
O2—H1*O*2⋯N1	0.82	1.86	2.602 (2)	149
C7—H7⋯O1	0.93	2.16	2.789 (3)	124

Symmetry code: (i) 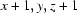.

**Fig. 1.**
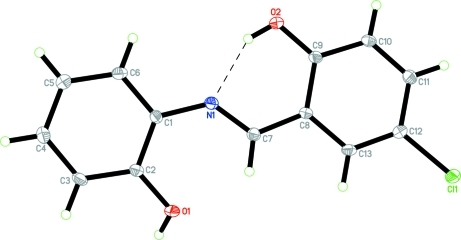
The molecular structure of the title compound, with 50% probability displacement ellipsoids and the atom-numbering scheme. The hydrogen bond is shown as a dashed line.

**Fig. 2.**
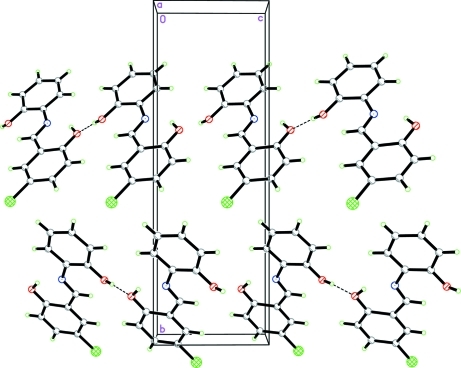
The crystal packing of the title compound viewed down the a axis, showing chains running along the [101] direction. Hydrogen bonds are shown as dashed lines.

## supplementary crystallographic information

### Comment

We have been interested in synthesis of Schiff base ligands and their complexes (Eltayeb *et al.*, 2007*a*,*b*) due to their applications such as analytical reagents for the determination of trace elements (Eltayeb & Ahmed, 2005*a*,*b*), pharmacological activities, anticancer (Dao *et al.*, 2000), anti-HIV (Sriram *et al.*, 2006), antibacterial and antifungal (Karthikeyan *et al.*, 2006) activities. The title compound was used to synthesis the chelated borate catalyst (Wei & Atwood, 1998). Herein we report the crystal structure of the title Schiff base ligand (I).

The molecule of (I) (Fig. 1), C_13_H_10_ClNO_2_, crystallizes in a *trans* configuration about the C═N bond [1.312 (3) Å] with a torsion angle C1–N1–C7–C8 = -179.2 (2)°. The molecule is almost planar with a dihedral angle between the two benzene rings being 2.48 (10)°. The chloro and two hydroxy groups lie on the same plane with their attached benzene rings with the r.m.s. of 0.0105 (2) Å for the seven non H atoms (C1, C2, C3, C4, C5, C6 and O1) and 0.0129 (2) Å for the eight non H atoms (C8, C9, C10, C11, C12, C13, O2 and Cl1). An intramolecular O—H···N hydrogen bond between the imine N atom and one hydroxy group generates an S(6) ring motif (Fig. 1 and Table 1) (Bernstein *et al.*, 1995). The bond distances are of normal values (Allen *et al.*, 1987) and are comparable with the related structure (Pu, 2008).

In the crystal packing (Fig. 2) , O—H···O hydrogen bonds (Table 1) which formed between the two hydroxy O atoms link the molecules into into chains along the [101] direction. The crystal is consolidated by O—H···O hydrogen bonds (Table 1) and C···Cl [3.584 (2)–3.646 (2) Å] short contacts.

### Experimental

The title compound was synthesized by adding 5-chloro-2-hydroxybenzaldehyde (0.312 g, 2 mmol) to the solution of 2-aminophenol (0.218 g, 2 mmol) in ethanol (30 ml). The mixture was refluxed with stirring for half an hour. The resultant yellow-orange solution was filtered and the filtrate was evaporated to give a yellow solid product. Yellow needle-shaped single crystals of the title compound suitable for *x*-ray structure determination were obtained from ethanol by slow evaporation at room temperature after a few days.

### Refinement

All H atoms were positioned geometrically and allowed to ride on their parent atoms, with *d*(O—H) = 0.82 Å and *d*(C—H) = 0.93 Å. The *U*_iso_(H) values were constrained to be 1.2*U*_eq_ of the carrier atoms. The highest residual electron density peak is located at 0.70 Å from C5 and the deepest hole is located at 0.05 Å from H1O2.

### Crystal data

**Table:**

C_13_H_10_ClNO_2_	*F*(000) = 256
*M**_r_* = 247.67	*D*_x_ = 1.533 Mg m^−^^3^
Monoclinic, *P*2_1_	Mo *K*α radiation, λ = 0.71073 Å
Hall symbol: P 2yb	Cell parameters from 2680 reflections
*a* = 4.6681 (9) Å	θ = 4.4–30.0°
*b* = 18.509 (3) Å	µ = 0.34 mm^−^^1^
*c* = 6.2118 (11) Å	*T* = 100 K
β = 90.980 (4)°	Needle, yellow
*V* = 536.63 (17) Å^3^	0.55 × 0.14 × 0.07 mm
*Z* = 2	

### Data collection

**Table:**

Bruker APEX Duo CCD area detector diffractometer	2680 independent reflections
Radiation source: sealed tube	2569 reflections with *I* > 2σ(*I*)
graphite	*R*_int_ = 0.023
φ and ω scans	θ_max_ = 30.0°, θ_min_ = 4.4°
Absorption correction: multi-scan (*SADABS*; Bruker, 2009)	*h* = −6→6
*T*_min_ = 0.834, *T*_max_ = 0.976	*k* = −24→26
4593 measured reflections	*l* = −8→8

### Refinement

**Table:**

Refinement on *F*^2^	Secondary atom site location: difference Fourier map
Least-squares matrix: full	Hydrogen site location: inferred from neighbouring sites
*R*[*F*^2^ > 2σ(*F*^2^)] = 0.034	H-atom parameters constrained
*wR*(*F*^2^) = 0.119	*w* = 1/[σ^2^(*F*_o_^2^) + (0.0756*P*)^2^ + 0.0404*P*] where *P* = (*F*_o_^2^ + 2*F*_c_^2^)/3
*S* = 1.20	(Δ/σ)_max_ = 0.001
2680 reflections	Δρ_max_ = 0.47 e Å^−^^3^
154 parameters	Δρ_min_ = −0.47 e Å^−^^3^
1 restraint	Absolute structure: Flack (1983), 1125 Friedel pairs
Primary atom site location: structure-invariant direct methods	Flack parameter: −0.03 (7)

### Special details

**Table:**

Experimental. The crystal was placed in the cold stream of an Oxford Cryosystems Cobra open-flow nitrogen cryostat (Cosier & Glazer, 1986) operating at 100.0 (1) K.
Geometry. All esds (except the esd in the dihedral angle between two l.s. planes) are estimated using the full covariance matrix. The cell esds are taken into account individually in the estimation of esds in distances, angles and torsion angles; correlations between esds in cell parameters are only used when they are defined by crystal symmetry. An approximate (isotropic) treatment of cell esds is used for estimating esds involving l.s. planes.
Refinement. Refinement of F^2^ against ALL reflections. The weighted R-factor wR and goodness of fit S are based on F^2^, conventional R-factors R are based on F, with F set to zero for negative F^2^. The threshold expression of F^2^ > 2sigma(F^2^) is used only for calculating R-factors(gt) etc. and is not relevant to the choice of reflections for refinement. R-factors based on F^2^ are statistically about twice as large as those based on F, and R- factors based on ALL data will be even larger.

### Fractional atomic coordinates and isotropic or equivalent isotropic displacement parameters (Å^2^)

**Table:**

	*x*	*y*	*z*	*U*_iso_*/*U*_eq_	
Cl1	0.02845 (11)	1.08595 (3)	0.36240 (8)	0.01689 (14)	
O1	1.1428 (4)	0.82828 (9)	0.5209 (2)	0.0167 (3)	
H1O1	1.2817	0.8379	0.5983	0.025*	
O2	0.5318 (4)	0.86922 (9)	−0.2123 (2)	0.0151 (3)	
H1O2	0.6550	0.8463	−0.1462	0.018*	
N1	0.8674 (4)	0.83076 (10)	0.1062 (3)	0.0116 (3)	
C1	1.0806 (4)	0.78021 (11)	0.1679 (3)	0.0113 (4)	
C2	1.2187 (4)	0.77923 (11)	0.3724 (3)	0.0119 (4)	
C3	1.4270 (5)	0.72625 (12)	0.4139 (3)	0.0144 (4)	
H3	1.5136	0.7236	0.5496	0.017*	
C4	1.5066 (5)	0.67719 (12)	0.2539 (4)	0.0157 (4)	
H4	1.6481	0.6430	0.2828	0.019*	
C5	1.3732 (5)	0.67964 (12)	0.0506 (4)	0.0147 (4)	
H5	1.4255	0.6471	−0.0559	0.018*	
C6	1.1629 (5)	0.73071 (12)	0.0089 (3)	0.0140 (4)	
H6	1.0747	0.7323	−0.1264	0.017*	
C7	0.7386 (5)	0.87972 (12)	0.2228 (3)	0.0122 (4)	
H7	0.7886	0.8845	0.3677	0.015*	
C8	0.5237 (5)	0.92579 (11)	0.1321 (3)	0.0114 (4)	
C9	0.4240 (5)	0.91833 (11)	−0.0859 (3)	0.0115 (4)	
C10	0.2019 (5)	0.96567 (12)	−0.1572 (3)	0.0132 (4)	
H10	0.1328	0.9622	−0.2981	0.016*	
C11	0.0865 (5)	1.01690 (12)	−0.0213 (3)	0.0136 (4)	
H11	−0.0572	1.0476	−0.0720	0.016*	
C12	0.1857 (5)	1.02276 (11)	0.1934 (3)	0.0127 (4)	
C13	0.4000 (5)	0.97834 (12)	0.2706 (3)	0.0124 (4)	
H13	0.4641	0.9825	0.4126	0.015*	

### Atomic displacement parameters (Å^2^)

**Table:**

	*U*^11^	*U*^22^	*U*^33^	*U*^12^	*U*^13^	*U*^23^
Cl1	0.0209 (3)	0.0156 (2)	0.0141 (2)	0.00493 (18)	−0.00206 (15)	−0.0037 (2)
O1	0.0172 (8)	0.0217 (8)	0.0111 (7)	0.0046 (6)	−0.0046 (5)	−0.0049 (6)
O2	0.0166 (8)	0.0178 (8)	0.0108 (7)	0.0039 (5)	−0.0021 (5)	−0.0036 (6)
N1	0.0124 (8)	0.0118 (8)	0.0106 (8)	−0.0001 (6)	−0.0010 (5)	−0.0005 (6)
C1	0.0108 (9)	0.0113 (9)	0.0116 (9)	0.0006 (7)	−0.0013 (6)	0.0001 (7)
C2	0.0120 (9)	0.0139 (9)	0.0098 (9)	−0.0003 (6)	−0.0006 (6)	0.0006 (7)
C3	0.0148 (10)	0.0187 (11)	0.0097 (9)	0.0017 (7)	−0.0025 (6)	0.0017 (8)
C4	0.0143 (10)	0.0143 (10)	0.0183 (11)	0.0024 (7)	−0.0012 (7)	0.0015 (8)
C5	0.0157 (10)	0.0145 (10)	0.0140 (10)	−0.0002 (7)	0.0012 (7)	−0.0013 (8)
C6	0.0152 (10)	0.0154 (10)	0.0113 (10)	−0.0010 (7)	−0.0011 (7)	−0.0025 (7)
C7	0.0117 (9)	0.0138 (9)	0.0111 (9)	0.0001 (6)	0.0000 (6)	0.0000 (7)
C8	0.0120 (9)	0.0128 (9)	0.0092 (9)	0.0002 (6)	−0.0012 (6)	−0.0003 (7)
C9	0.0126 (10)	0.0130 (9)	0.0090 (9)	0.0001 (7)	−0.0002 (6)	0.0004 (7)
C10	0.0155 (10)	0.0157 (9)	0.0083 (9)	0.0009 (7)	−0.0011 (7)	0.0016 (7)
C11	0.0142 (10)	0.0134 (9)	0.0132 (10)	0.0006 (7)	−0.0008 (6)	0.0016 (8)
C12	0.0143 (10)	0.0121 (9)	0.0117 (9)	−0.0007 (7)	0.0017 (6)	−0.0012 (8)
C13	0.0138 (10)	0.0155 (10)	0.0077 (9)	−0.0011 (7)	−0.0001 (6)	−0.0005 (7)

### Geometric parameters (Å, °)

**Table:**

Cl1—C12	1.742 (2)	C5—C6	1.384 (3)
O1—C2	1.346 (3)	C5—H5	0.9300
O1—H1O1	0.8200	C6—H6	0.9300
O2—C9	1.308 (3)	C7—C8	1.425 (3)
O2—H1O2	0.8200	C7—H7	0.9300
N1—C7	1.312 (3)	C8—C13	1.427 (3)
N1—C1	1.414 (3)	C8—C9	1.431 (3)
C1—C6	1.405 (3)	C9—C10	1.422 (3)
C1—C2	1.415 (3)	C10—C11	1.385 (3)
C2—C3	1.402 (3)	C10—H10	0.9300
C3—C4	1.401 (3)	C11—C12	1.408 (3)
C3—H3	0.9300	C11—H11	0.9300
C4—C5	1.399 (3)	C12—C13	1.375 (3)
C4—H4	0.9300	C13—H13	0.9300
			
C2—O1—H1O1	109.5	N1—C7—C8	121.39 (19)
C9—O2—H1O2	109.5	N1—C7—H7	119.3
C7—N1—C1	129.38 (18)	C8—C7—H7	119.3
C6—C1—N1	116.14 (18)	C7—C8—C13	117.29 (18)
C6—C1—C2	119.79 (18)	C7—C8—C9	122.13 (19)
N1—C1—C2	124.01 (19)	C13—C8—C9	120.52 (18)
O1—C2—C3	122.39 (18)	O2—C9—C10	121.83 (18)
O1—C2—C1	119.04 (18)	O2—C9—C8	120.86 (18)
C3—C2—C1	118.56 (19)	C10—C9—C8	117.30 (18)
C4—C3—C2	120.96 (19)	C11—C10—C9	121.41 (18)
C4—C3—H3	119.5	C11—C10—H10	119.3
C2—C3—H3	119.5	C9—C10—H10	119.3
C5—C4—C3	120.0 (2)	C10—C11—C12	120.33 (19)
C5—C4—H4	120.0	C10—C11—H11	119.8
C3—C4—H4	120.0	C12—C11—H11	119.8
C6—C5—C4	119.6 (2)	C13—C12—C11	120.7 (2)
C6—C5—H5	120.2	C13—C12—Cl1	120.17 (16)
C4—C5—H5	120.2	C11—C12—Cl1	119.16 (17)
C5—C6—C1	121.0 (2)	C12—C13—C8	119.77 (19)
C5—C6—H6	119.5	C12—C13—H13	120.1
C1—C6—H6	119.5	C8—C13—H13	120.1
			
C7—N1—C1—C6	175.5 (2)	N1—C7—C8—C9	4.0 (3)
C7—N1—C1—C2	−7.2 (4)	C7—C8—C9—O2	−1.3 (3)
C6—C1—C2—O1	178.20 (19)	C13—C8—C9—O2	−178.44 (19)
N1—C1—C2—O1	1.0 (3)	C7—C8—C9—C10	177.9 (2)
C6—C1—C2—C3	−2.8 (3)	C13—C8—C9—C10	0.8 (3)
N1—C1—C2—C3	180.0 (2)	O2—C9—C10—C11	179.1 (2)
O1—C2—C3—C4	−178.3 (2)	C8—C9—C10—C11	−0.1 (3)
C1—C2—C3—C4	2.8 (3)	C9—C10—C11—C12	−0.6 (3)
C2—C3—C4—C5	−1.4 (3)	C10—C11—C12—C13	0.6 (3)
C3—C4—C5—C6	0.0 (3)	C10—C11—C12—Cl1	−178.08 (17)
C4—C5—C6—C1	−0.1 (3)	C11—C12—C13—C8	0.1 (3)
N1—C1—C6—C5	178.9 (2)	Cl1—C12—C13—C8	178.79 (15)
C2—C1—C6—C5	1.5 (3)	C7—C8—C13—C12	−178.1 (2)
C1—N1—C7—C8	−179.2 (2)	C9—C8—C13—C12	−0.8 (3)
N1—C7—C8—C13	−178.77 (19)		

### Hydrogen-bond geometry (Å, °)

**Table:**

*D*—H···*A*	*D*—H	H···*A*	*D*···*A*	*D*—H···*A*
O1—H1O1···O2^i^	0.82	1.74	2.553 (2)	169
O2—H1O2···N1	0.82	1.86	2.602 (2)	149
C7—H7···O1	0.93	2.16	2.789 (3)	124

Symmetry codes: (i) *x*+1, *y*, *z*+1.
